# Clinical and laboratory characteristics of solitary rectal ulcer syndrome: a retrospective analysis of 36 case

**DOI:** 10.1038/s41598-025-86324-0

**Published:** 2025-01-16

**Authors:** Furkan Ali Uygur, Muhammed Emin Göktepe, Gökhan Aydın, Enes Ağırman, Ahmet Cumhur Dülger

**Affiliations:** 1 Faculty of Medicine, Department of General Surgery, Giresun University, Gazipaşa Yerleşkesi Debboy Mevkii P.K., 28200 Merkez-GİRESUN, Turkey; 2 Department of Family Medicine, Görele Op. Dr. Ergun Ozdemir State Hospital, Görele, Turkey; 3 Faculty of Medicine, Department of Gastroenterology, Giresun University, Giresun, Turkey; 4 Department of General Surgery, Erzurum City Hospital, Erzurum, Turkey

**Keywords:** Colorectal disorder, SRUS, Hematochezia, Ulcerative colitis, Endoscopic submucosal dissection, Diseases, Signs and symptoms

## Abstract

The primary objective of this study was to evaluate the clinical, laboratory, and histological characteristics of solitary rectal ulcer syndrome (SRUS) and assess the outcomes associated with various management strategies. This retrospective observational study was conducted at Giresun Education and Research Hospital. This study included patients diagnosed with SRUS between January 2020 and January 2024. Demographic information, clinical presentation, primary diagnosis, and laboratory parameters were obtained from electronic medical records. Statistical analysis was performed using SPSS software, and the chi-square test was used to compare categorical variables. A total of 36 patients diagnosed with SRUS were included, with the majority being male (80.6%), and the mean age of participants was 75.6 years. Hematochezia was identified as the most common initial symptom (61.1%), followed by abdominal pain (16.7%) and constipation (11.1%). Laboratory findings revealed significant abnormalities, including mean hemoglobin levels of 10.0 ± 2.4 g/dL and mean CRP levels of 56.7 ± 65.4 mg/L. Histopathological analysis showed that 38.9% of patients had normal biopsy results, whereas inflammation and dysplasia were observed in 41.7% and 2.8% of cases, respectively. Additionally, a statistically significant difference in age was observed between the patients presenting with different initial symptoms (*p* = 0.028). The study also found that biopsy results varied significantly across symptom groups (*p* = 0.012), and although differences in hemoglobin and hematocrit levels across biopsy groups were noted, they were not statistically significant. This study offers a comprehensive analysis of SRUS, emphasizing the importance of detailed clinical and laboratory evaluation. Hematochezia emerged as the most prevalent symptom, and ulcerative colitis was identified as the primary diagnosis. Significant associations were observed between various clinical parameters and patient outcomes, highlighting the necessity of a multidisciplinary approach in managing SRUS.

## Introduction

Solitary rectal ulcer syndrome (SRUS) is a rare and often misunderstood rectal disorder, first described by Cruveilhier in 1829 and later extensively characterized by Madigan and Morson in 1969. SRUS typically manifests with a constellation of symptoms including rectal bleeding, mucus discharge, straining during defecation, and a sensation of incomplete evacuation^1,2^. Despite its name, the condition may present with multiple ulcers or even polypoid and erythematous lesions, rather than a solitary ulcer. The pathogenesis of SRUS is multifactorial, with key contributing factors, including rectal prolapse, paradoxical contraction of the puborectalis muscle, and direct trauma from straining or digital manipulation^3^.

The diagnosis of SRUS presents a significant challenge owing to its varied presentation and overlap with other gastrointestinal conditions, such as inflammatory bowel disease, infectious proctitis, and ischemic colitis. Endoscopic examination typically reveals ulceration, erythema, and polypoid lesions, predominantly located on the anterior rectal wall. Histopathologically, SRUS is characterized by fibromuscular obliteration of the lamina propria, crypt distortion, and inflammatory infiltrates, distinguishing it from other colonic pathologies^4,5^. Solitary rectal ulcers can arise from various gastrointestinal conditions other than SRUS, making differential diagnosis essential^6^. Conditions such as inflammatory bowel disease, ischemic colitis, and infectious proctitis may present with solitary or multiple rectal ulcers that mimic SRUS^7^. These conditions often have overlapping symptoms, including rectal bleeding and mucosal ulceration, but can be differentiated by specific histopathological findings and clinical presentations unique to each disorder.

The management of SRUS requires a multidisciplinary approach, beginning with conservative measures, such as dietary modification, bowel training, and biofeedback therapy aimed at correcting defecatory dysfunction. For patients who do not respond to these initial interventions, surgical options including rectopexy, mucosal resection, and fecal diversion may be necessary in severe cases^3,5^.

This article provides a comprehensive analysis of 36 cases of solitary rectal ulcers identified in the endoscopy unit of the Giresun Education and Research Hospital between January 2020 and January 2023. By examining the clinical features, diagnostic approaches, treatment modalities, and outcomes of these cases, we aimed to enhance our understanding and management of this complex condition.

## Materials and methods

### Study design

This study was a retrospective observational analysis conducted at Giresun Education and Research Hospital. Patients diagnosed with SRUS who were admitted to the endoscopy unit between January 2020 and January 2024. The primary objective of this study was to evaluate the clinical, laboratory, and histological characteristics of SRUS and assess the outcomes associated with various management strategies.

### Data collection

Data were retrieved from the hospital’s electronic medical records, including demographic information (age, sex, and blood type), clinical presentation (primary complaints), and primary diagnosis. Laboratory parameters included white blood cell count (WBC), hemoglobin (HGB), hematocrit (HCT), glucose, urea, creatinine, glomerular filtration rate (GFR), alanine transaminase (ALT), aspartate transaminase (AST), total bilirubin (TBIL), direct bilirubin (DBIL), indirect bilirubin (IND.BIL), lactate dehydrogenase (LDH), amylase, and C-reactive protein (CRP). Serological markers included anti-HIV and anti-HBs antibodies. Imaging studies included abdominal ultrasonography (USG) of the liver, gallbladder, spleen, and kidneys. A histopathological analysis was also performed.

### Diagnostic criteria

SRUS diagnosis was established based on a combination of clinical symptoms, endoscopic findings, and histopathological confirmation. Clinical symptoms include rectal bleeding, mucus discharge, straining during defecation, and a sensation of incomplete evacuation. Endoscopic examination revealed ulceration, erythema, or polypoid lesions predominantly located on the anterior rectal wall. Histopathological analysis confirmed the diagnosis by demonstrating fibromuscular obliteration of the lamina propria, crypt distortion, and inflammatory infiltration.

### Treatment modalities and follow-up

Thirty patients were administered rectal mesalamine at a dose of 1 g twice daily for 15 days. At the 6-month follow-up, among the 23 patients who returned for evaluation, 19 showed no evidence of SRUS lesions, while persistent lesions were observed in 4 patients. Additionally, two patients presenting with rectal bleeding were treated with argon plasma coagulation (APC), achieving complete remission in both cases. Four patients received rectal steroid suppositories. Furthermore, all patients were advised to adopt a high-fiber diet to support bowel health and improve symptom management. This additional information provides a comprehensive overview of treatment strategies and observed outcomes, thereby enhancing the understanding of SRUS management.

### Ethical considerations

The study protocol was approved by the Institutional Review Board of Giresun Education and Research Hospital (27.03.2023/09-KAEK-71). Due to the retrospective nature of this study, the requirement for informed consent was explicitly waived by the Institutional Review Board of Giresun Education and Research Hospital. Patient confidentiality was maintained by anonymizing all data, and all procedures were conducted in accordance with the principles outlined in the Declaration of Helsinki.

### Statistical analyses

Statistical analyses were conducted using SPSS software (version 24.0; IBM Corp., Armonk, NY, USA). Descriptive statistics were used to summarize the demographic and clinical characteristics. Continuous variables were presented as mean ± standard deviation (SD) or median (interquartile range), while categorical variables were expressed as frequencies and percentages. The chi-square test was used to compare categorical variables. Statistical significance was defined as *p* < 0.05.

## Results

A total of 36 patients diagnosed with SRUS were included in the study. The majority of the participants were male, with 29 males accounting for 80.6% of the sample. The mean age of participants was 75.6 years with a standard deviation of 15.4 years. Regarding blood type distribution, 3.0% of the participants had blood type O Rh (-), 30.3% had blood type O Rh (+), 3.0% had blood type A Rh (-), 39.4% had blood type A Rh (+), 3.0% had blood type AB Rh (+), and 21.2% had blood type B Rh (+). Hematochezia was reported as the first symptom by 61.1% of participants, while constipation was the first symptom in 11.1% of cases. Abdominal pain was reported as the first symptom in 16.7% of the participants, and other symptoms were the first symptom in 11.1% of the cases (Table 1).

**Table 1:** Demographic and clinical characteristics of the study participants.

		Mean±S.D./Count (%)
Gender (Male)		29 (80.6%)
Age (year)		75.6±15.4
Blood type	0 Rh (-)	1 (3.0%)
	0 Rh (+)	10 (30.3%)
	A Rh (-)	1 (3.0%)
	A Rh (+)	13 (39.4%)
	AB Rh (+)	1 (3.0%)
	B Rh (+)	7 (21.2%)
First symptom	Hematochezia	22 (61.1%)
	Constipation	4 (11.1%)
	Abdominal pain	6 (16.7%)
	Other symptoms	4 (11.1%)

The mean white blood cell count (WBC) was 9.3 ± 5.0 × 10^3^/µL. The mean hemoglobin (HGB) level was 10.0 ± 2.4 g/dL, and the mean hematocrit (HCT) was 31.7 ± 6.3%. The mean glucose level was 124.1 ± 46.0 mg/dL. The mean urea level was 78.8 ± 63.4 mg/dL, and the mean creatinine level was 1.1 ± 0.8 mg/dL. The glomerular filtration rate (GFR) had a mean value of 65.7 ± 28.1 mL/min/1.73 m^2^. The mean alanine aminotransferase (ALT) level was 18.7 ± 20.7 U/L, and the mean aspartate aminotransferase (AST) level was 35.5 ± 81.7 U/L. The mean total bilirubin level was 0.5 ± 0.3 mg/dL, with direct bilirubin at 0.3 ± 0.3 mg/dL and indirect bilirubin at 0.2 ± 0.1 mg/dL. The mean lactate dehydrogenase (LDH) level was 291.5 ± 278.3 U/L. The mean amylase level was 71.8 ± 65.8 U/L. The mean calcium level was 8.8 ± 1.2 mg/dL. The mean sodium level was 140.7 ± 5.8 mmol/L, chloride was 103.3 ± 6.8 mmol/L, and potassium was 4.3 ± 0.8 mmol/L. The mean C-reactive protein (CRP) level was 56.7 ± 65.4 mg/L. The mean thyroid-stimulating hormone (TSH) level was 2.0 ± 2.3 mIU/L, and the mean thyroxine (T4) level was 1.2 ± 0.3 µg/dL (Table 2).

**Table 2:** Laboratory parameters of the study participants.

	Mean±S.D.
WBC (10^3^/µL)	9.3±5.0
HGB (g/dL)	10.0±2.4
HTC (%)	31.7±6.3
Glukoz (mg/dL)	124.1±46.0
BUN (mg/dL)	78.8±63.4
Creatin (mg/dL)	1.1±0.8
GFR (mL/dak/1.73 m^2^)	65.7±28.1
ALT (U/L)	18.7±20.7
AST (U/L)	35.5±81.7
Total bilirubin (mg/dL)	0.5±0.3
Direct bilirubin (mg/dL)	0.3±0.3
Indirect bilirubin (mg/dL)	0.2±0.1
LDH (U/L)	291.5±278.3
Amilaz (U/L)	71.8±65.8
Calcium (mg/dL)	8.8±1.2
Sodium (mmol/L)	140.7±5.8
Clor (mmol/L)	103.3±6.8
Potassium (mmol/L)	4.3±0.8
CRP (mg/L)	56.7±65.4
TSH (mIU/L)	2.0±2.3
T4 (µg/dL)	1.2±0.3

Of the study participants, 38.9% had normal biopsy results. Inflammation was observed in 41.7% of the patients. Dysplasia alone was found in 2.8% of the participants, whereas a combination of inflammation and dysplasia was present in 16.7% of the cases (Table 3).

**Table 3:** Rectal Ulcer Biopsy results of the study participants.

		Count	Column *N*%
Rectal ulcer biopsy	Normal	14	38.9%
	Inflammation	15	41.7%
	Dysplasia	1	2.8%
	Inflammation + Dysplasia	6	16.7%

The mean age of participants who presented with hematochezia was 81.1 ± 10.4 years. Those who presented with constipation had a mean age of 61.8 ± 15.3 years, while the mean age for those with abdominal pain was 63.3 ± 16.2 years. Participants with other symptoms had a mean age of 77.3 ± 23.8 years. The differences in age between these groups were statistically significant, with a p-value of 0.028 (Table 4).

**Table 4:** Comparison of Age among participants based on initial symptoms.

	Hemotochesia (*n* = 22)	Constipation (*n* = 4)	Abdominal pain (*n* = 6)	Other symptoms (*n* = 4)	
	Mean±S.D.	Mean±S.D.	Mean±S.D.	Mean±S.D.	p value
Age (year)	81.1±10.4	61.8±15.3	63.3±16.2	77.3±23.8	**0.028**

Among the participants with hematochezia, 45.5% had normal biopsy results, while none of those with constipation or abdominal pain had normal results. All the participants with other symptoms had normal biopsy results (100%). Inflammation was observed in 36.4% of participants with hematochezia, 50% of those with constipation, and 83.3% of those with abdominal pain, and none of the participants had other symptoms. Dysplasia was present in 25% of the participants with constipation, while no dysplasia was observed in those with hematochezia, abdominal pain, or other symptoms. A combination of inflammation and dysplasia was found in 18.2% of participants with hematochezia, 25% of those with constipation, and 16.7% of those with abdominal pain, and none of the participants had other symptoms. The differences in biopsy results across the symptom groups were statistically significant (*p* = 0.012 (Table 5).

**Table 5:** Rectal Ulcer Biopsy results based on initial symptoms.

		Hemotochesia (*n* = 22)	Constipation (*n* = 4)	Abdominal pain (*n* = 6)	Other symptoms (*n* = 4)	
		Count (%)	Count (%)	Count (%)	Count (%)	*p* value
Rectal ulcer biopsy	Normal	10 (45.5)	0 (0)	0 (0)	4 (100)	0.012
	Inflammation	8 (36.4)	2 (50)	5 (83.3)	0 (0)	
	Dysplasia	0 (0)	1 (25)	0 (0)	0 (0)	
	Inflammation + Dysplasia	4 (18.2)	1 (25)	1 (16.7)	0 (0)	

Participants with normal biopsy results had a mean age of 82.6 ± 14.3 years, while those with inflammation had a mean age of 68.9 ± 16.3 years. The single participant with dysplasia had an age of 81.0 years, and those with both inflammation and dysplasia had a mean age of 75.0 ± 10.8 years. The differences in age between these groups approached statistical signife with (*p* = 0.079). The mean hemoglobin (HGB) level was 9.8 ± 2.1 g/dL in participants with normal biopsy results, 10.1 ± 3.0 g/dL in those with inflammation, 8.7 g/dL in the participant with dysplasia, and 10.7 ± 1.8 g/dL in those with both inflammation and dysplasia. The differences in hemoglobin levels among these groups were not statistically significant (*p* = 0.801). The mean hematocrit (HTC) was 30.7 ± 6.2% in participants with normal biopsy results, 32.2 ± 7.0% in those with inflammation, 28.7% in those with dysplasia, and 33.3 ± 5.6% in those with both inflammation and dysplasia. The differences in the hematocrit levels were also not statistically significant (*p* = 0.804) (Table 6).

**Table 6:** Comparison of Age, Hemoglobin, and hematocrit levels based on rectal Ulcer Biopsy results.

	Normal (*n* = 14)	Inflammation (*n* = 15)	Dysplasia (*n* = 1)	Inf.+Dsysplasia (*n* = 6)	
	Mean±S.D.	Mean±S.D.	Mean±S.D.	Mean±S.D.	*p* değeri
Age (year)	82.6±14.3	68.9±16.3	81.0±.	75.0±10.8	0.079
HGB (g/dL)	9.8±2.1	10.1±3.0	8.7±.	10.7±1.8	0.801
HTC (%)	30.7±6.2	32.2±7.0	28.7±.	33.3±5.6	0.804

Analysis of the relationship between blood groups and histopathological findings revealed no statistically significant association (chi-square test, *p* > 0.05). Table 7 illustrates the distribution of histopathological results across different blood groups (Table 7).

**Table 7:** Association between blood groups and histopathological findings.

Blood group	Normal findings	Abnormal findings	*p* value
0 Rh-	0 (%0)	1 (%100)	> 0.05
0 Rh+	4 (%40)	6 (%60)	
A Rh-	0 (%0)	1 (%100)	
A Rh+	6 (%46)	7 (%54)	
AB Rh+	1 (%100)	0 (%0)	
B Rh+	2 (%29)	5 (%71)	

In the current study, three distinct treatment modalities were employed for patients diagnosed with solitary rectal ulcer syndrome (SRUS): rectal mesalamine, argon plasma coagulation (APC), and rectal steroid suppositories. Thirty patients received rectal mesalamine (1 g twice daily) for 15 days. At the 6-month follow-up, among the 23 patients who returned for evaluation, 19 showed complete resolution of SRUS lesions, while four had persistent lesions. For patients presenting with rectal bleeding, APC was applied to two individuals, both of whom achieved complete remission. Additionally, rectal steroid suppositories were administered to four patients as part of conservative management. All patients were advised to adopt a high-fiber diet to enhance bowel health and effectively manage symptoms. The follow-up outcomes indicated that rectal mesalamine and APC were particularly effective in achieving lesion resolution, with APC providing rapid relief in patients with active bleeding.

## Discussion

The diagnostic challenge of SRUS is well documented, with many ulcerative or hyperemic lesions often misidentified as inflammatory bowel diseases. A key histological feature that distinguishes SRUS from these other conditions is the obliteration of fibromuscular tissue in the lamina propria, which serves as a highly sensitive marker. Overactivity of the anal sphincter has been recognized as a significant pathophysiological factor that contributes to increased intrarectal pressure, elevated transmural gradient, and heightened voiding pressure, ultimately resulting in venous congestion and ulceration. Our analysis did not demonstrate a significant association between the blood groups and the histopathological findings of SRUS. This suggests that the blood type may not be a contributing factor to the development or severity of SRUS. To the best of our knowledge, there is limited literature exploring this relationship. Therefore, our findings contribute new information to the field, indicating that blood grouping may not need to be considered when assessing the SRUS risk or prognosis.

In our study, age differences were observed based on biopsy results, with patients with normal biopsy results having a higher mean age. This finding suggests the potential impact of age on the histopathological characteristics of SRUS. Similarly, in the study by Abid et al., the mean age of patients was reported as 37.4 years^8^, indicating that SRUS is generally diagnosed in younger individuals. However, the higher prevalence of normal biopsy results in the older age group in our study highlights the possible influence of age on histopathological findings of SRUS.

Additionally, the term “solitary rectal ulcer syndrome” may not fully encompass the diverse clinical presentations of this condition, which can include polypoidal, nodular, multiple ulcers, hyperplastic polyps, erythematous lesions, and telangiectasias. A more inclusive nomenclature could help better categorize and understand the variations under SRUS diagnosis, thereby enhancing diagnostic accuracy and informing more targeted therapeutic strategies^9^.

Consistent with previous studies^5,8,10^, our findings showed that hematochezia was the most prevalent symptom among SRUS patients with SRUS. For instance, Ejaz et al.^10^ and Shafiq^5^ reported hematochezia as the most common presenting complaint in their cohort. Additionally, we observed a predominance of SRUS in older adults with a mean age of 75.6 years, in contrast to the study by Zhu et al.^11^, which reported a higher incidence of SRUS in younger adults in the third and fourth decades of life. This discrepancy may be attributed to regional differences, lifestyle factors, or variations in the population demographics. The findings of this study are consistent with those reported in previous SRUS studies^5,8,10^, which highlighted the challenges in diagnosing SRUS owing to its varied presentation and the importance of considering it in the differential diagnosis of rectal bleeding, especially in older patients. This consistency aligns with our observations, particularly the high prevalence of hematochezia and predominance of SRUS in older adults, underscoring the common clinical presentation of SRUS.

A significant finding in our study was the prevalence of ulcerative colitis as a primary diagnosis in 77.78% of patients. This is higher than that reported in other studies, such as those by Park et al. and Uza et al., who identified SRUS predominantly in patients with chronic constipation and rectal prolapse but did not emphasize ulcerative colitis as a major associated condition^12,13^. This discrepancy could be attributed to regional differences or variations in the diagnostic criteria. Correlation analysis revealed several important relationships between the clinical parameters and outcomes. Age was positively correlated with urea and CRP levels, suggesting that older patients may experience higher levels of inflammation and kidney function abnormalities. This finding is consistent with the literature, where age-related changes in kidney function and elevated inflammatory markers have been well documented^14^.

In our study, the significant age differences among the symptom groups indicated that older patients were more likely to present with hematochezia. This may be attributed to age-related vascular fragility and comorbid conditions, which increase the risk of bleeding. The variation in biopsy results across different symptom groups suggests that the clinical presentation of SRUS is heterogeneous and may influence the histopathological findings. The lack of statistically significant differences in hemoglobin and hematocrit levels across the biopsy groups implies that anemia may not be directly correlated with the histopathological severity of SRUS. These findings highlight the importance of patient age and symptomatology in the diagnosis and management of SRUS.

Our ultrasonographic findings indicated that liver abnormalities such as hepatosteatosis and cysts were common in patients with SRUS. Histologically, inflammation was present in 47.22% of patients, and dysplasia was observed in 13.89%. These findings align with those of a study by Abid et al., which reported inflammation and fibromuscular obliteration as common histological features of SRUS^15^.

Our findings on rectal mesalamine’s effectiveness in SRUS align with those of AlGhulayqah et al., who observed a significant improvement in 85% of SRUS patients treated with mesalamine and a high-fiber diet. In our study, 82.6% of the patients showed complete lesion resolution with rectal mesalamine at the six-month follow-up, supporting its role as an effective therapy^16^. Additionally, we applied APC in two cases with active rectal bleeding, both achieving complete remission, similar to Shah et al., who reported 100% bleeding control and 71% ulcer healing in APC-treated refractory SRUS patients. This suggests that combining mesalamine and APC may offer dual benefits: mesalamine for inflammation reduction and APC for immediate bleeding control^17^. These findings underscore the potential of mesalamine and APC as effective complementary options for SRUS management, particularly for patients with persistent symptoms or active bleeding.

The clinical characteristics and findings of our case series underscore the rarity and chronic nature of SRUS, with an incidence of 1 per 100,000 adults. Although this condition is benign, it is frequently misdiagnosed, with up to 26% of patients having an incorrect initial diagnosis. We hope that our findings will help to reemphasize the presence of SRUS in the literature, thereby aiding clinicians in maintaining awareness and improving diagnostic accuracy. These insights should encourage more comprehensive and multicenter studies to further elucidate the complexities of SRUS and to optimize patient outcomes.

### Limitations

This study has several limitations, including its retrospective design and relatively small sample size. Furthermore, as the study was conducted at a single center, the generalizability of the findings may be limited. Another notable limitation is the absence of colonoscopic data for most patients at the time of diagnosis. The endoscopic evaluations predominantly focused on targeted procedures, such as rectoscopy or sigmoidoscopy, and no prior screening or colonoscopic interventions were documented before the diagnosis of solitary rectal ulcer syndrome (SRUS). Additionally, biofeedback therapy, which is recognized as an important treatment modality for SRUS, was not administered to any patients in this study. This decision was made to ensure homogeneity in the patient population and to minimize variability due to differences in therapeutic interventions. However, the lack of biofeedback therapy may limit the applicability of our findings to centers where such interventions are routinely performed. Future prospective studies incorporating biofeedback therapy are warranted to evaluate its impact on SRUS management and outcomes.

## Conclusion

This study offers a comprehensive analysis of SRUS and highlights its clinical, laboratory, and histological characteristics. Hematochezia was identified as the most common symptom, with ulcerative colitis being the predominant primary diagnosis. Significant correlations were observed between various clinical parameters and patient outcomes, underscoring the need for a multidisciplinary approach for managing SRUS. These findings contribute to the existing literature and may help guide future research and clinical practice regarding SRUS diagnosis and treatment.

## Data Availability

The datasets used and/or analyzed during the current study are available from the corresponding author upon reasonable request.
